# Gall Bladder Perforation as a Complication of Typhoid Fever

**DOI:** 10.4103/1319-3767.43282

**Published:** 2008-10

**Authors:** Anand Pandey, Ajay N. Gangopadhyay, Vijayendra Kumar

**Affiliations:** Department of Pediatric Surgery, Institute of Medical Sciences, Banaras Hindu University, Varanasi-221 005, U.P., India. E-mail: gangulybhu@rediffmail.com

Sir,

Among various described complications of typhoid fever, acute cholecystitis is a rare one,[1] while perforation of the gallbladder is extremely uncommon. We share our experience of gall bladder perforation—this rare complication of typhoid. A 10-year-old boy presented to us with fever of 10 days' duration, distension of the abdomen, and an inability to pass flatus and feces for two days. Clinical examination demonstrated the presence of guarding, rigidity, and rebound tenderness. An X-ray of the abdomen showed multiple air fluid levels, but no free air. The Widal test was strongly positive for Salmonella typhi ‘O’ and S. typhi ‘H’ but negative for S. paratyphi.

After initial resuscitation, the patient was operated; exploratory laparotomy revealed a bile-stained abdomen with matted bowel loops. There was no bowel perforation, but a large perforation in its fundus was noticed on exploring the gall bladder. Cholecystectomy was preformed; the postoperative period was uneventful, and the patient was discharged on the 10th postoperative day.

Acute cholecystitis is a rare complication of typhoid,[1] presenting mostly in the first week of illness. Characteristic findings include fever, abdominal pain, diarrhea, vomiting, jaundice, and a palpable mass.[1] The clinical features suggestive of gall bladder perforation are nonspecific. Paracentesis may reveal bile-stained ascitic fluid.[2] Abdominal X-rays may not show pneumoperitoneum as seen in our patient, and hence, they are not always helpful. Ultrasonography and computerized tomography may demonstrate abdominal fluid but lack specificity to diagnose gall bladder perforation, which can be easily detected on hepatobiliary scanning.[3] A high index of suspicion is needed to diagnose the condition. Surgical options include cholecystostomy or cholecystectomy.[1] However, we believe that cholecystectomy may be desirable to prevent the carrier state of typhoid fever.

Perforation of the gall bladder may be caused by an inflammatory reaction and weakness of its wall in the course of the disease. Histology shows inflammatory changes in the gall bladder[2] that were also noticed in our patient.

Gall bladder perforation after typhoid cholecystitis is an uncommon occurrence in a pediatric population. A simple literature search revealed fewer than 10 cases in children, and all these reports were very old. 

Gall bladder perforation in the setting of typhoid requires a high degree of clinical suspicion, especially in the pediatric population. Hence, surgeons must bear in mind the possibility of this complication while treating any pediatric patient presenting with a history of prolonged fever and signs of peritonitis. Cholecystectomy is the treatment of choice with a reasonable outcome.
